# Low adherence to exercise may have influenced the proportion of OMERACT-OARSI responders in an integrated osteoarthritis care model: secondary analyses from a cluster-randomised stepped-wedge trial

**DOI:** 10.1186/s12891-020-03235-z

**Published:** 2020-04-13

**Authors:** Tuva Moseng, Hanne Dagfinrud, Leti van Bodegom-Vos, Krysia Dziedzic, Kåre Birger Hagen, Bård Natvig, Jan Harald Røtterud, Thea Vliet Vlieland, Nina Østerås

**Affiliations:** 1 National Advisory Unit on Rehabilitation in Rheumatology, Department of Rheumatology, Diakonhjemmet Hospital, P.O. Box 23 Vinderen, N-0319 Oslo, Norway; 2 Department of Biomedical Data Sciences, Leiden University Medical Center, Leiden, The Netherlands; 3 School for Primary, Community and Social Care, Primary Care Centre Versus Arthritis, Keele University, Keele, UK; 4 Department of General Practice, Institute of Health and Society, University of Oslo, Oslo, Norway; 5 Department of Orthopaedic Surgery, Akershus University Hospital, Lørenskog, Norway; 6 Department of Orthopaedics, Leiden University Medical Center, Leiden, The Netherlands

**Keywords:** Osteoarthritis, Hip, Knee, Management, RCT, Exercise, Pain, Function, Responder, Adherence

## Abstract

**Background:**

To address the well-documented gap between hip and knee osteoarthritis (OA) treatment recommendations and current clinical practice, a structured model for integrated OA care was developed and evaluated in a stepped-wedge cluster-randomised controlled trial. The current study used secondary outcomes to evaluate clinically important response to treatment through the Outcome Measures in Rheumatology Clinical Trials clinical responder criteria (OMERACT-OARSI responder criteria) after 3 and 6 months between patients receiving the structured OA care model vs. usual care. Secondly, the study aimed to investigate if the proportion of responders in the intervention group was influenced by adherence to the exercise program inherent in the model.

**Methods:**

The study was conducted in primary healthcare in six Norwegian municipalities. General practitioners and physiotherapists received training in OA treatment recommendations and use of the structured model. The intervention group attended a physiotherapist-led OA education program and performed individually tailored exercises for 8–12 weeks. The control group received usual care. Patient-reported pain, function and global assessment of disease activity during the last week were evaluated using 11-point numeric rating scales (NRS 0–10). These scores were used to calculate the proportion of OMERACT-OARSI responders. Two-level mixed logistic regression models were fitted to investigate differences in responders between the intervention and control group.

**Results:**

Two hundred eighty-four intervention and 109 control group participants with hip and knee OA recruited from primary care in six Norwegian municipalities. In total 47% of the intervention and 35% of the control group participants were responders at 3 or 6 months combined; showing an uncertain between-group difference (OR_adjusted_ 1.38 (95% CI 0.41, 4.67). In the intervention group, 184 participants completed the exercise programme (exercised ≥2 times/week for ≥8 weeks) and 55% of these were classified as responders. In contrast, 28% of the 86 non-completers were classified as responders.

**Conclusions:**

The difference in proportion of OMERACT-OARSI responders at 3 and 6 months between the intervention and control group was uncertain. In the intervention group, a larger proportion of responders were seen among the exercise completers compared to the non-completers.

**Clinical trial registration:**

Clinicaltrials.gov identifier: NCT02333656. Registered 7. January 2015.

## Background

There is a well-documented gap between evidence and practice in the management of patients with hip and knee osteoarthritis [1, 2]. The situation calls for development and evaluation of effective OA management programmes in order to enhance the quality of OA care and improve patient outcomes.

The international research consortium “Joint Effort Initiative” has defined an OA management program as: *a model of evidence-based, non-surgical OA care implemented in a real-world clinical setting* [3]. Such models should, according to this definition, incorporate: i) personalised, tailored care; ii) provided as a care package with reassessments and progression; iii) incorporate at least two of the three first-line treatments (patient education, exercise and weight management); and iv) include optional evidence-based adjunctive treatments (e.g. braces, assistive devices).

We have conducted a cluster randomized controlled stepped-wedge trial to evaluate the effectiveness of implementing a structured model of integrated OA care in six Norwegian municipalities [4]. The previously reported primary outcome: patient-reported quality of care and associated hypotheses showed greater quality of care and satisfaction with care, more patients referred to physiotherapy and fewer to orthopedic surgeons, and more patients who fulfilled physical activity criteria in the intervention group compared to the usual care group after 6 months [5]. Another publication from this study has further reported improved uptake of the first-line treatments in the intervention group compared to the control group, but low adherence regarding exercise adherence in the intervention group [6].

To our knowledge, only one other previous study has used a randomised controlled design (RCT) to test the effectiveness of an OA management program in primary care [7]. While this study reported increased uptake of the core NICE OA recommendations, no between-group differences were found for any of the patient-reported outcomes on OA related pain and function [8]. In addition, a handful of other OA management programmes, employing less rigorous, observational study designs have previously been set up in a limited number of countries [9, 10]. Generally, these programmes report somewhat diverging, but overall promising effects on pain and physical function [11–13]. In summary, more high-quality studies to evaluate effects of OA management programmes in primary care are currently warranted.

It represents a common challenge for all previous management programmes to reproduce the consistent effects on patient-reported outcomes as found in the more stringent exercise RCTs, which informs the treatment recommendations [14, 15]. As management programmes are complex, there are multitudes of possible reasons for this. In order to design programs that effectively influence OA patients’ symptoms, it is vital to disentangle some of this complexity. Such investigation can identify and evaluate the importance of certain key components of a programme. One such component can be programmes’ specific strategies to improve exercise adherence. High adherence is suggested to be essential to achieve the optimal symptom-modifying effects from exercise for hip and knee OA [16]. The direct relationship between exercise adherence and patient-reported outcomes has not yet been investigated in any of the previous OA management programmes.[4]

The aim of the current secondary analyses (as predefined in the study protocol) was to explore patient-reported response to treatment between the intervention and control group including application of the Outcome Measures in Rheumatology Clinical Trials clinical responder criteria (OMERACT-OARSI responder criteria) at 3 and 6 months follow-up combined. Additionally, we aimed to further explore the intervention group by: i) Comparing the proportion of OMERACT-OARSI responders among participants completing the exercise programme (exercise for ≥2 times per week for ≥8 weeks) vs. the proportion among the non-completers; ii) Examine demographic and baseline patient-reported measures for associations with completing the exercise programme.

## Methods

### Design

**Fig. 1:**
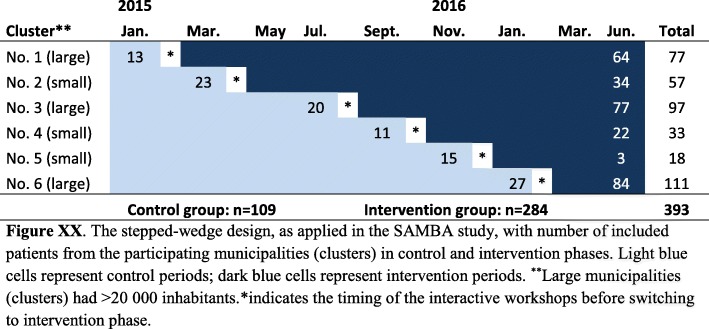
The stepped-wedge cluster randomized design as applied for the SAMBA study

The study was a cluster-randomised controlled trial, conducted with a stepped-wedge cohort design in six neighbouring Norwegian municipalities (clusters) between January 2015 and October 2017. The study involved general practitioners (GPs), physiotherapists (PTs) and patients with symptomatic hip and knee OA. In the stepped-wedge design all clusters started the trial simultaneously in control phase, before switching to intervention phase in a randomised order based on pre-defined time points. The design is explained in detail in Fig. 1.

The study protocol has been published previously [4]. The study is reported according to the CONSORT checklist (Additional file 2).

According to the stepped-wedge design the six municipalities (clusters) were randomly assigned to one of the six sequences for time of cross-over from control to intervention phase using a computer-generated list of random numbers provided by a statistician. To ensure a mix of municipality sizes in the randomised sequence, stratification on the number of inhabitants (less than versus more than 20,000) was performed. Due to the nature of the intervention, it was not possible to blind the involved GPs, PTs or patients.

Patient-reported outcomes were collected at baseline at 3 and 6 month’s follow-up, using electronic questionnaires.

### Participants

All GPs and all PTs working in private practice or in a Healthy Life Centre in the six municipalities were invited to participate in the study. Potential patients eligible for the study were identified by their GP or PT with the following inclusion criteria: age ≥ 45 years with activity-related hip and/or knee pain/complaints and clinical signs and symptoms corresponding to hip and/or knee OA or radiologically diagnosed OA or registered in the medical journal with a hip or knee OA diagnosis.

Patients who did not understand the Norwegian language; had undergone joint replacement for all affected hip/knee OA joints; or had inflammatory rheumatic diseases, malignant illness or other major conditions that restricted their ability to adhere to the treatment recommendations were excluded. Patient participants included during the control phases constituted the control group, whereas patient participants included during the intervention phases constituted the intervention group.

### Intervention

The SAMBA model for integrated OA care (Additional file 1, Fig. A) was developed by the research team and included a structured pathway for the patient through the health care system. “SAMBA” is the acronym for the Norwegian study title, “Collaboration for improved OA care.”

The model should provide the patient with access to timely recommended care.

Interactive workshops for GPs and PTs were the main activity to ensure implementation of the SAMBA model in clinical practice. The workshops were arranged separately for each municipality, in close proximity to the time for crossover from control to intervention phase. During the workshops, the PTs received education in the delivery of the standardized patient-education programme and of the individually tailored exercise programme. The PTs received access to a database with recommended exercises and dose recommendations. The exercises were selected from the exercise programmes of the Swedish BOA and Danish GLA:D OA management programmes and previously published well-recognized, effective exercise programmes to impact hip and knee OA symptoms [4, 17, 18] .

The group-based patient education programme lasted 3 h and was based on a standardized template (Power-Point with optional manuscript). The patient education focused on providing the patient with knowledge of OA and recommend treatments, emphasizing the importance of exercise.

Following individual patient examinations, the PTs prescribed individually tailored exercise programmes with the main aim to improve muscular strength, in order to reduce OA symptoms. The PTs were instructed to closely monitor the individual patients’ performance and to regularly adjust exercise dose and degree of difficulty to facilitate progression. Dose recommendations were based on acknowledged, international guidelines from American College of Sports Medicine (ACSM), and included gradually increasing the dose towards 2–4 sets with 8–12 repetitions of 60 - 70% of 1 repetition maximum or more if tolerated [19]. The PTs were instructed to increase the resistance when the patient could perform 2 extra repetitions in the last set (“The 2+ principle”).

The patients performed their individual exercise programmes in groups of 5–10 patients per PT. The exercise period lasted 8–12 weeks, with two supervised weekly sessions. The patients were encouraged to add a third home-based session consisting of 30–60 min cardiorespiratory exercise like brisk walking, running or bicycling. Patients who did not wish to attend the group sessions had the option of performing their exercise individually, but were expected to consult their PT for regular adjustments of the programme.

During the control phase, the GPs and PTs provided usual care for their OA patients. Usual care can be heterogeneous in nature and could include any treatment the GP and patient considered appropriate. Physiotherapy (all kind of modalities) was allowed, but not provided by a PT who had participated in the workshops.

### Outcome measures

Patient-reported outcomes were evaluated at 3 and 6 months. These included pain, physical function, stiffness and patient global assessment of disease activity during the last week evaluated using 11-point Numeric Rating Scales (NRS) (0 = best, 10 = worst). Hip/knee related quality of life was evaluated with the Hip disability and Osteoarthritis Outcome Score (HOOS) and Knee injury and Osteoarthritis Outcome Score (KOOS), quality of life subscale, (0–100, 100 = no problems). Daily hours spent in sitting position was evaluated with one question asking: “*how many hours do you usually spend in sitting position during a regular day*”. The scores from the pain, function and patient global assessment of disease activity outcomes were used to calculate the proportion of OMERACT-OARSI responders [20] (in the two groups) at 3 and 6 months. A participant was classified as a responder if one of the following were fulfilled:

1) High improvement in pain or function

- ≥50% improvement + absolute change of ≥2 in pain, OR
- ≥50% improvement + absolute change of ≥2 in function

2) Improvement in at least two of the three following:

- ≥20% improvement + absolute change ≥1 in pain
- ≥20% improvement + absolute change ≥1 in function
- ≥20% improvement + absolute change ≥1 in the patient global assessment of disease activity

A participant was characterized as having completed the exercise programme if having exercised for ≥2 times per week for ≥8 weeks. The information on the number of completed exercise sessions was derived from participants’ exercise diaries and attendance lists from the PTs if exercise diaries were not returned or incomplete.

### Statistical analysis

Sample size calculations were not conducted for secondary outcomes of the study. For the main outcome, it was calculated a need for a total of 388 participants [4].

Descriptive analyses were conducted for baseline characteristics.

The difference in pain, function, disease activity, stiffness, HOOS/KOOS quality of life, daily hours in sitting position and proportion of OMERACT-OARSI responders between the intervention and control group were investigated using two-level mixed regression models with random effects for cluster and individual participant and fixed effects for age, gender, BMI and time trends (month of inclusion) on an intention to treat basis. The linear models for pain, function, disease activity, HOOS/KOOS quality of life and daily hours in sitting position included an interaction term of follow-up time point and group. The difference in OMERACT-OARSI responders at 3 and 6 months was investigated using a logistic model. The proportion of responders was investigated separately for 3 and 6 months and for these time-points combined. These models account for missing under a missing at random assumption.

In the intervention group, the difference in the proportion of responders between the participants completing the exercise programme (exercise for ≥2 times per week for ≥8 weeks) and the non-completers were compared using descriptive statistics.

To investigate potential associations between completing the exercise programme and baseline characteristics a two-level mixed logistic regression was fitted. The model included random effects for cluster and individual participant. The set of baseline characteristics (fixed effects) were selected based on experience and previous literature of variables suggested to have an impact on exercise adherence using a fit full model approach. The selected variables were age, gender, BMI, education (< 1 / ≥ 1 year of university), employment (yes/no), cohabitation (yes/no), multisite OA (yes/no), pain last week (NRS 0–10) and self-efficacy (Arthritis Self-Efficacy Scale (ASES) mean score of Pain and Function subscales (10–100, “very uncertain” to “certain”) [21]. The model was additionally adjusted for time trends (month of inclusion), investigated for possible multicollinearity and the goodness of fit using Hosmer and Lemeshow test.

The significance level for the analyses was set at *p* < 0.05.

### Patient and public involvement

Two patient research partners were members of the trial steering group and involved in all stages of this trial including grant application, development of study material (patient information, consent procedures, questionnaires), intervention, interpretation and dissemination of the results.

## Results

**Fig. 2:**
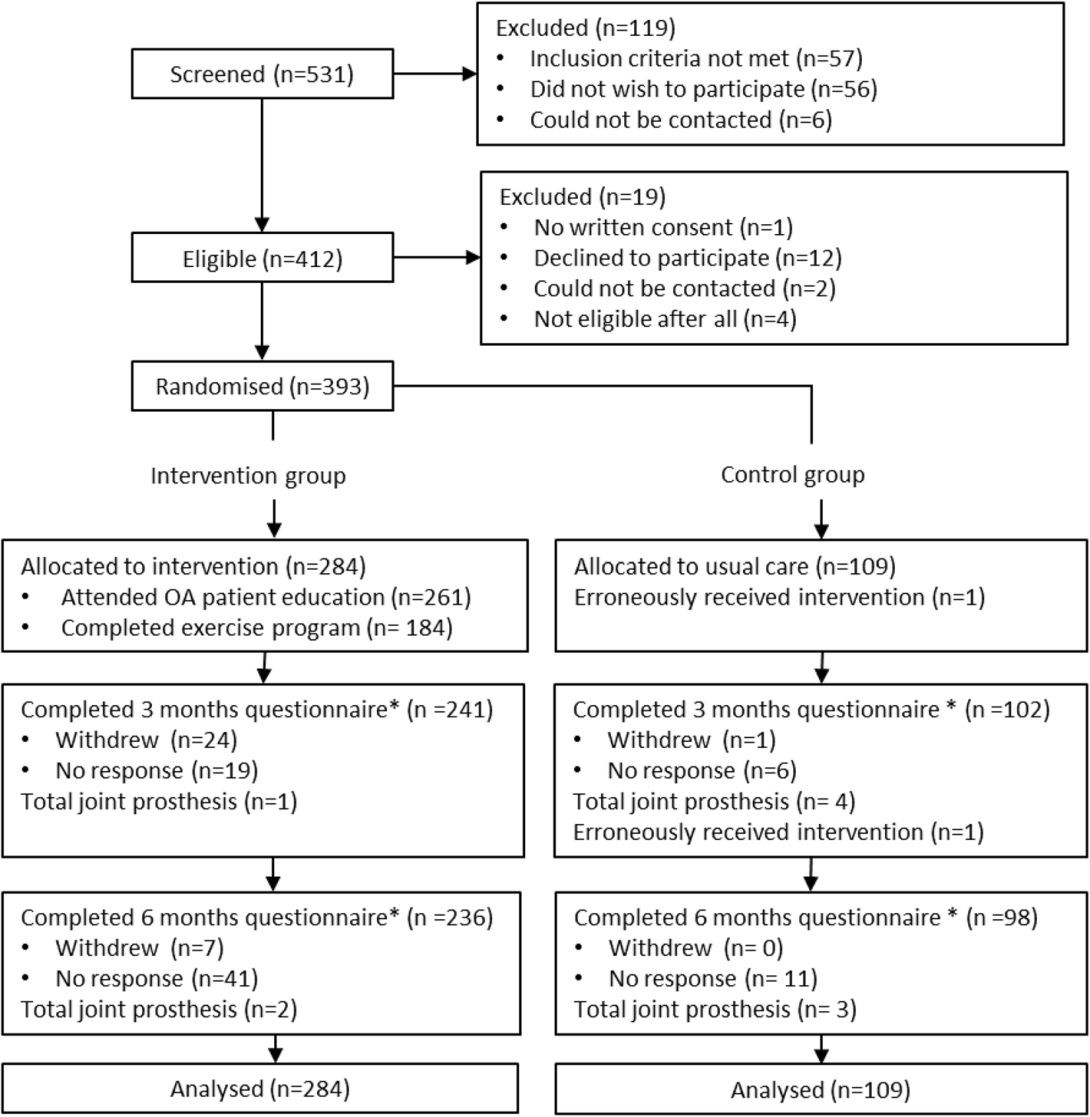
Flowchart of participants’ progress through the phases of the trial. *Refers to the questions regarding pain, function and disease activity

A total of 393 patients with hip and/or knee OA were included, 109 during the control phases (=control group) and 284 during the intervention phases (=intervention group). Figure 2 illustrates the flow of participants through the study and provides reasons for ineligibility or exclusion. Baseline characteristics of the participants are displayed in Table 1.

**Table 1:** Baseline characteristics of patients with osteoarthritis participating in the SAMBA study

	All participants	Intervention group	Control group
	(*n* = 393)	(*n* = 284)	(*n* = 109)
Age, mean (SD)	63 (10)	63 (10)	65 (10)
Sex, female, n (%)	280 (71)	211 (74)	68 (62)
BMI, mean (SD)	29 (6)	29 (6)	28 (5)
Education			
≥ 1 year university, n (%)	136 (35)	101 (36)	35 (32)
Main affected joint, n (%)			
Knee	228 (58)	174 (61)	54 (49)
Hip	146 (37)	100 (35)	46 (42)
Other*	18 (6)	9 (3)	9 (8)
Multi-site OA, n (%)**	339 (86)	244 (86)	95 (87)

*OA joint other than hip and knee (e.g. hand, ankle) is the main affected joint ** ≥ 2 affected joints

In total 88% (*n* = 348) completed the patient-reported questionnaires at 3 months, and 88% (*n* = 345) at 6 months follow-up (Fig. 2). Those not responding to the questionnaires at 3 or 6 months were similar to those responding with regard to baseline characteristics. Seven (6%) OA patients in the control group and 3 (1%) in the intervention group received joint replacement surgery between baseline and the 6-month follow-up.

In the intervention group, 65% (*n* = 184) exercised ≥2 times per week for ≥8 weeks. Four patients in the intervention group experienced increased prolonged knee pain and/or swelling and discontinued the exercise programme at the halfway stage. Of the 102 included control group participants, 43% self-reported having received physiotherapy once or more between baseline and 6 months.

### Pain, function, disease activity and OMERACT-OARSI responders

The mean differences in the patient-reported outcomes and proportion of OMERACT-OARSI responders at 3 and 6 months are presented in Table 2. At both 3 and 6 months the intervention group reported marginally less pain, disease activity, and improved function compared to the control group on the 0–10 scale. Similar results were also seen for H/KOOS quality of life subscale, with marginally higher scores in the intervention group and slightly less time spent in sitting position at 3 and 6 months.

**Table 2:** Differences in patient-reported outcomes and OMERACT-OARSI responders between the intervention and control group at 3 and 6 months follow-up

	Intervention group				Control group				Control vs. intervention 3 mos. (95% CI)	*p*	Control vs. intervention 6 mos. (95% CI)	*p*	Control vs. Intervention group 3 + 6 mos. (95% CI)	*p*
	BL *n* = 283	3 mos. *n* = 242	6 mos. *n* = 239	3 + 6 mos.	BL n = 109	3 mos. *n* = 106	6 mos. *n* = 106	3 + 6 mos.						
Pain last week (NRS 0–10), mean (SD)	5.4 (2.0)	4.4 (2.0)	4.2 (2.1)		5.1 (1.8)	4.7 (2.2)	4.7 (2.1)		b: −0.65 (−1.26, −0.04)	0.04	b: − 0.98 (− 1.59, − 0.37)	0.002		
Function last week (NRS 0–10), mean (SD)	5.2 (2.0)	4.4 (1.9)	4.1 (2.1)		4.9 (1.9)	4.6 (2.3)	4.7 (2.1)		b: −0.67 (−1.28, − 0.06)	0.03	b: − 1.17 (− 1.78, − 0.56)	< 0.001		
Disease activity last week (NRS 0–10), mean (SD)	5.3 (2.0)	4.3 (2.0)	4.2 (2.1)		4.8 (2.0)	4.7 (2.3)	4.7 (2.2)		b: −0.93 (−1.55, − 0.31)	0.003	b: − 1.02 (− 1.64, − 0.39)	0.001		
OMERACT-OARSI responders, n (%)		92 (33)	99 (35)	132 (47)		24 (23)	24 (23)	36 (35)	OR: 1.37 (0.26, 7.24)	0.7	OR: 2.81 (0.32, 24.67)	0.3	OR: 1.38 (0.41, 4.67)	0.6
Stiffness last week (NRS 0–10), mean (SD)	5.3 (2.3)	4.5 (2.1)	4.3 (2.1)		4.9 (2.2)	4.6 (2.1)	4.9 (2.1)		b: −0.63 (−1.28, 0.01)	0.05	b: − 1.10 (− 1.74, − 0.45)	0.001		
H/KOOS QoL subscale mean (SD)	44.9 (16.3)	47.8 (14.9)	49.7 (15.8)		49.9 (17.2)	45.3 (18.2)	47.2 (17.5)		b: 5.43 (0.59, 10.27)	0.03	b: 5.11 (0.28, 9.95)	0.04		
Daily hours in sitting position, mean (SD)	6.5 (2.8)	6.1 (2.8)	5.9 (2.6)		6.8 (3.6)	6.4 (3.2)	6.2 (3.0)		b:-1.17 (−2.04, −0.31)	0.008	b: −1.47 (− 2.33, − 0.60)	0.001		

*b* Beta. *OR* Odds Ratio. Estimates are adjusted for patient age, sex, BMI and study months (number of months between study initiation and the patients’ baseline questionnaire). H/KOOS QoL = Hip disability and Osteoarthritis Outcome Score/ Knee injury and Osteoarthritis Outcome Score, Quality of Life subscale, (0–100. 100 = no problems)

When applying the follow-up data to the OMERACT-OARSI responder criteria the proportion of responders at 3 months was 33% (*n* = 92) in the intervention group compared to 23% (*n* = 24) in the control group. At 6 months we found 35% (*n* = 99) responders in the intervention group compared to 23% (*n* = 24) responders in the control group. When the total number of responders at 3 and 6 months was combined 47% (*n* = 132) in the intervention group and 35% (*n* = 36) in the control group were classified as responders.

### OMERACT-OARSI responders and adherence to the exercise programme (intervention group)

**Fig. 3:**
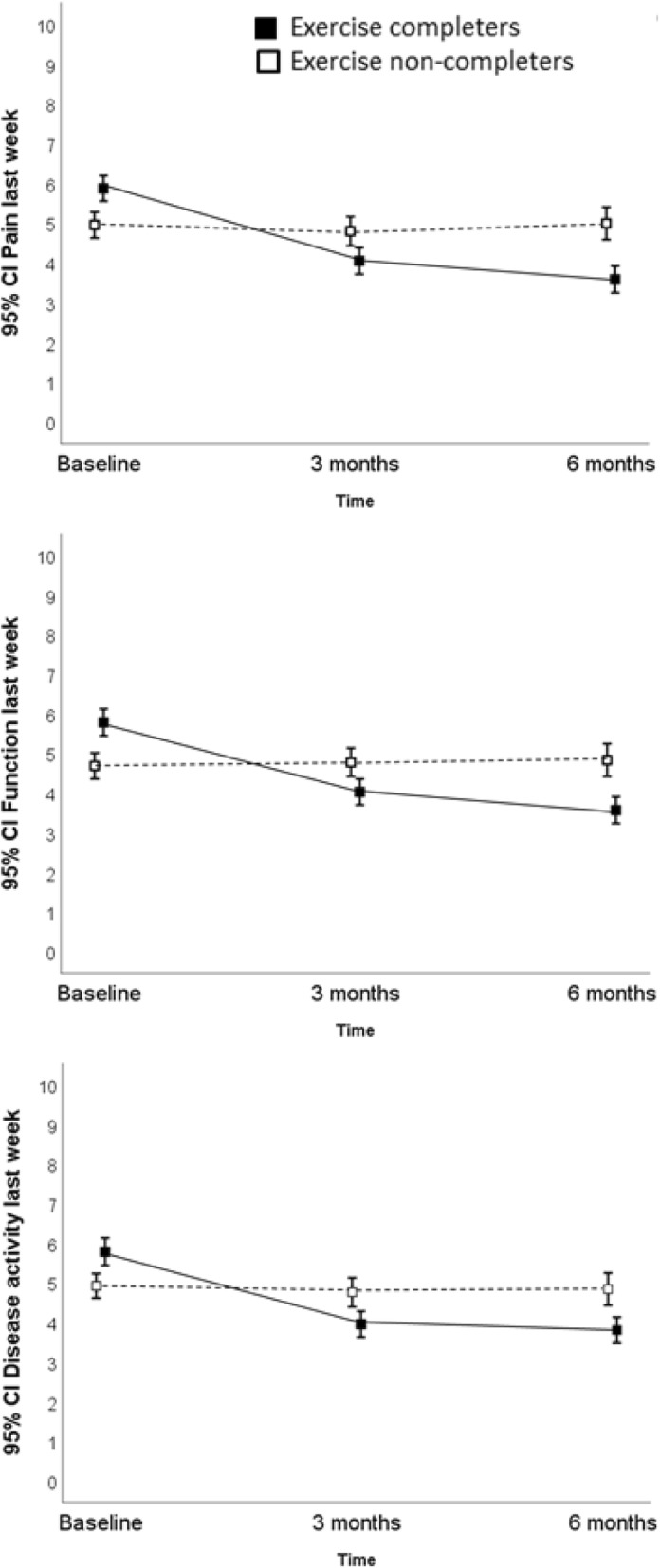
Difference in OA related pain, function and disease activity between exercise program completers (exercised ≥2 times per week for ≥8 weeks) and non-completers

Of the 184 intervention group patients who completed the exercise programme 55% (*n* = 101) were classified as OMERACT-OARSI responders at 3 or 6 months combined. For the 86 patients who did not complete the exercise programme, 28% (*n* = 24) were classified as OMERACT-OARSI responders at 3 or 6 months combined. Data were missing from 14 participants. Differences in pain, physical function and disease activity between the exercise programme completers and non-completers at baseline and 3 and 6 months follow-up are displayed in Fig. 3. The figure indicates that the participants completing the exercise programme report more baseline symptoms, but also have a more beneficial symptom trajectory from baseline to 3 and 6 months compared to the non-completers.

### Factors associated with completing the exercise programme (intervention group)

Results of the logistic regression analysis to explore measures associated with completing the exercise programme are displayed in Table 3. The variables multisite OA and employment were removed from the model due to multicollinearity. In this model none of the selected variables was significantly associated with completing the exercise programme.

**Table 3:** Association between exercise programme completers and selected baseline variables

Variable	Completer *n* = 184	Non-Completer *n* = 86	Adjusted OR (95% CI)	*p*
Age, mean (SD)	63.4 (9.5)	61.3 (9.8)	1.02 (0.98, 1.05)	0.2
Sex, female, n (%)	141 (77.0)	62 (72.1)	1.60 (0.86, 3.00)	0.1
BMI, mean (SD)	28.5 (5.5)	29.8 (5.9)	0.97 (0.92, 1.02)	0.2
Education, ≥ 1 year university, n (%)	65 (35.7)	28 (32.9)	1.07 (0.60, 1.91)	0.8
Living with someone, n (%)	144 (78.7)	66 (77.6)	1.25 (0.63, 2.45)	0.5
Self-efficacy (10 worst-100 best), mean (SD)	57.9 (17.6)	55.5 (21.1)	1.01 (0.99, 1.02)	0.4
Pain (NRS 0–10), mean (SD)	5.2 (1.9)	5.7 (2.1)	0.94 (0.80, 1.09)	0.4

The model includes random effects of cluster and individual participant and fixed effects for the other included variables and with additional adjustments for time (month of inclusion). OR = Odds ratio

## Discussion

Only small differences in mean pain, function, stiffness, disease activity, hip/knee related quality of life and hours spent sitting were observed between the intervention and control group at 3 and 6 months follow-up. The estimated difference in the proportion of participants fulfilling the OMERACT-OARSI responder criteria between the intervention and control group at 3 and 6 months combined was uncertain. A larger proportion of the intervention group participants who completed the exercise programme fulfilled the OMERACT-OARSI responder criteria compared to the non-completers. No demographic or baseline patient-reported measures were significantly associated with completing the exercise programme among the intervention group participants. The results indicate differences in the trajectories of pain, physical function and disease activity between exercise programme completers and non-completers.

These results display some of the challenges and complexities of implementing OA treatment recommendations in real-world clinical practice. In this study, we have targeted the structure of care delivery, educated health professionals and provided readily available tools to ease the delivery of recommended treatment alternatives [4]. Unfortunately, this intervention was not sufficient to show a clinically meaningful difference in patient-reported outcomes and a statistically significant difference in proportion of OMERACT-OARSI responders in the intervention group compared to the usual care control group. However, the total proportion of responder in the intervention group (47%) is similar to previous RCTs applying the OMERACT-OARSI responder criteria to evaluate the effect of different physiotherapist led exercise interventions on knee OA [22–24].

As much as 43% of the control group participants reported receiving at least one session of physiotherapy between baseline and 6 months. This could have influenced the relative high proportion of responders in the control group (35%) and thus contributed to the non-significant difference in proportion of responders between the intervention and control group.

When investigating the intervention group further, the proportion of responders was higher among the participants completing the exercise programme (55%) compared to the non-completers (28%). We believe this result highlights that adherence to exercise is an important component which needs to be thoroughly addressed to achieve the best possible results on pain and physical function in OA management programmes [25, 26]. In contrast, a recent study investigating adherence to a home-exercise programme for patients with knee OA was unable to show any relationship between exercise adherence and pain and self-reported function [27]. Yet, the authors were only able to use exercise frequency as a measure of adherence, which is the similar measure we have applied in the current analyses. In a separate paper reporting from the SAMBA study, we showed that a large proportion of the patients who exercised according to recommended frequency did not follow the recommendations for intensity, nor received the recommended progression in their exercise programmes [6]. We do, however recognize the weakness of this comparison as we have only used descriptive statistics. Outcomes such as psychological factors and comorbidities might be confounding factors in the relationship between responders and exercise adherence [28]. However, we decided not to perform advanced statistics on subset of data from the current RCT, due to the a high risk of bias associated with such analyses [29]. Two recent systematic reviews have given evidence that not only exercise frequency but also exercise intensity is important to impact OA symptoms in hip and knee OA [30–32]. The combination of these findings suggests that the proportion of responders in the intervention group could have been higher if more participants followed the ACSM recommendations on both exercise frequency and intensity [19]. However, improved adherence to exercise in clinical practice is not easily achieved. Previous studies have found small effects of certain behaviour change techniques and booster sessions to improve exercise adherence among patients [33, 34]. How to effectively improve exercise adherence in patients with hip and knee OA is a current research priority [35], which should be further studied also within the current setting. Patient motivation and beliefs may be important factors to consider [36].v

None of the demographic or baseline patient-reported measures were significantly associated with completing the exercise programme among the intervention group participants. A previous study has pointed to low-income and no baseline pain with pivoting and twisting as predictors for non-adherence to exercise in patients with meniscal tears and knee osteoarthritis [37]. Our data also indicates that participants not completing the exercise programme have a lower symptom burden at baseline compared to the completers. It is however, unknown if the participants completing the exercise program had a better response due to the higher symptom burden at baseline or due to completing the exercise program. For future studies it is relevant to examine this relationship further.

### Strengths and limitations

A major strength of this study is the randomised controlled design, combined with the broad inclusion criteria and pragmatic set-up. This combination provides a high external validity of the results toward real-life clinical practice. The usual care control group made it possible to form valid conclusions of the effectiveness of the SAMBA model regarding difference in proportion of responders between the intervention and control group. Another strength was the application of the OMERACT-OARSI responder criteria which is a recommended tool to assess change in OA symptoms after non-pharmacological interventions both in research and clinical practice [38].

Our study also holds some limitations which should be considered. Firstly, the analyses were conducted from secondary outcomes of an RCT. Power calculations were not performed for these outcomes. Secondly, our measure of adherence was a mix between data from patients’ self-reported exercise diaries and the physiotherapists’ attendance lists. It is known that people tend to overestimate exercise behaviour [39]. On the other hand, it is also possible that participants would sometimes forget to record their exercise sessions. Patients could also have exercised at home, which the PTs would not be able to document on their attendance lists. It is therefore a risk that exercise adherence could be both over- and underestimated. Due to this limitation we chose not to conduct a more sophisticated statistical analysis on the influence of exercise adherence on the proportion of responders. Methods such as a complier average causal effect analysis could have been appropriate if such methods had been developed for cluster-randomized stepped-wedge trials. Thirdly, the size of the groups is unbalanced due to a higher recruitment rate during the intervention phases of the study. As potential patient participants were identified by their GP and PT, it is possible that a recruitment bias exists.

## Conclusions

To conclude, we found an uncertain difference between the proportion of OMERACT-OARSI responders in the intervention and control group at 3 and 6 months combined. In the intervention group, the proportion of responders was higher among the completers of the exercise programme vs. the non-completers. None of the selected demographic or patient-reported baseline variables was associated with completing the exercise programme among the intervention group participants. Hence, exercise adherence may be an important contributing factor to achieve patient-reported effects within OA care models in primary care.

## Supplementary information

**Additional file 1 Figure A**. The SAMBA model for integrated OA care

**Additional file 2.** Modified CONSORT 2010 checklist of information to include when reporting a randomised trial with extensions for stepped-wedge design

## Data Availability

The datasets generated and analysed during the current study are not publicly available due to privacy regulations but are available from the corresponding author on reasonable request.

## Abbreviations

OA: Osteoarthritis
NRS: Numeric Rating Scale
OR: Odds Ratio
CI: Confidence Interval
RCT: Randomized Controlled Trial
GP: General Practitioner
PT: Physiotherapist
ACSM: American College of Sports Medicine
ASES: Arthritis Self-Efficacy Scale
MD: Mean Difference
