# Supplementary figures and images for: Low adherence to exercise may have influenced the proportion of OMERACT-OARSI responders in an integrated osteoarthritis care model: secondary analyses from a cluster-randomised stepped-wedge trial
Source: BMC Musculoskelet Disord. 2020 Apr 13;21:236. doi: 10.1186/s12891-020-03235-z (PMC7155273; doi:10.1186/s12891-020-03235-z)

**Additional file 1**


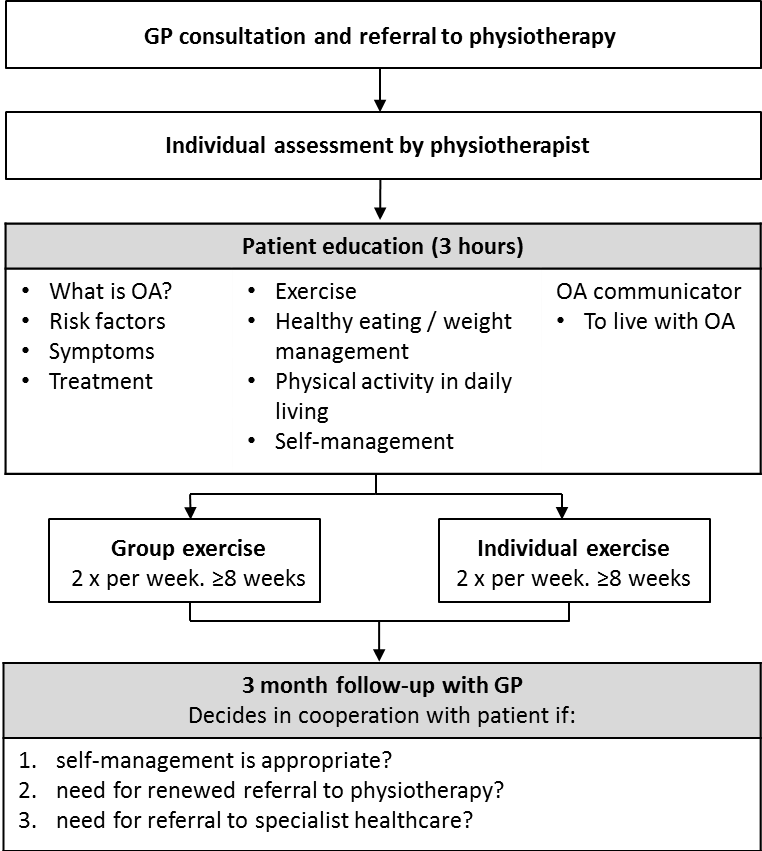


**Figure A.** The SAMBA model for integrated OA care

Supplement: Supplementary file 1 — Additional file 1 Figure A. The SAMBA model for integrated OA care [file 12891_2020_3235_MOESM1_ESM.docx]
